# The relationship between subclinical hypothyroidism and invasive papillary thyroid cancer

**DOI:** 10.3389/fendo.2023.1294441

**Published:** 2023-12-20

**Authors:** Changlin Li, Jiao Zhang, Gianlorenzo Dionigi, Hui Sun

**Affiliations:** ^1^ Division of Thyroid Surgery, China-Japan Union Hospital of Jilin University, Jilin Provincial Key Laboratory of Surgical Translational Medicine, Jilin Provincial Engineering Laboratory of Thyroid Disease Prevention and Control, Changchun, Jilin, China; ^2^ Division of Surgery, Istituto Auxologico Italiano, Istituto di Ricovero e Cura a Carattere Scientifco (IRCCS), Milan, Italy; ^3^ Department of Medical Biotechnology and Translational Medicine, University of Milan, Milan, Italy

**Keywords:** subclinical hypothyroidism, papillary thyroid cancer, extrathyroidal extension, body mass, autophagy

## Abstract

**Background:**

Subclinical hypothyroidism is the most common thyroid dysfunction. Approximately 10% of patients with thyroid cancer have subclinical hypothyroidism. There is a paucity of real-world studies examining the relationship between subclinical hypothyroidism and known correlates of invasiveness of papillary thyroid carcinoma (PTC).

**Materials and methods:**

A retrospective cohort study of 13,717 patients with PTC was conducted. Odds ratios were calculated to assess the relationship between subclinical hypothyroidism and extrathyroidal extension (ETE) after adjusting for BMI and genders. The Cancer Genome Atlas (TCGA) data were utilized for the analysis of TSHR-associated pathways, while qRT-PCR was employed to validate the expression levels of pivotal genes in the relevant signaling pathways.

**Results:**

In total, 13,717 PTC patients (10,769 women and 2,948 men; mean [SD] age, 42.90 [9.43] years) were included in the retrospective study. Subclinical hypothyroidism was an independent risk factor for ETE (OR adjusted, 1.168 [95% CI, 1.028–1.327]; *P*=0.017). In normal-weight patients, subclinical hypothyroidism was an independent risk factor for ETE (OR adjusted, 1.287 [95% CI, 1.089–1.520]; *P*=0.003). However, this risk was not observed in under-weight, overweight, and obese patients. Compared to females, subclinical hypothyroidism was a higher risk factor for ETE in male patients with normal body weight (OR male=2.363 vs. OR female=1.228). Subclinical hypothyroidism was found to be a significant risk factor for ETE in the subgroup of patients younger than 38 years old (OR1 adjusted, 1.382 [95% CI, 1.032–1.852], *P*=0.030). The findings from Gene Set Enrichment Analysis (GSEA) and Kyoto Encyclopedia of Genes and Genomes (KEGG) enrichment analysis revealed the involvement of the autophagy signaling pathway in TSHR/ETE/EMT regulation. Moreover, the gene expression levels demonstrated a concentration-dependent relationship between TSH intervention levels and the expression of key genes in the autophagy pathway of thyroid cancer cells.

**Conclusion:**

Subclinical hypothyroidism was an independent risk factor for ETE in patients with PTC. This association was particularly significant in normal-weight and younger patients. The risk of ETE associated with subclinical hypothyroidism was higher in males compared to females. Our study indicates a potential involvement of the autophagy pathway in regulating the ETE phenotype in thyroid cancer, specifically in the context of subclinical hypothyroidism.

## Introduction

In China, the incidence of papillary thyroid carcinoma (PTC), the most common endocrine cancer worldwide, has steadily increased over the past two decades (1). Several conditions are associated with PTC, including family history of goiter, exposure to high radiation levels, and certain hereditary syndromes (2, 3). In addition, obesity and subclinical hypothyroidism have been shown to be associated with thyroid cancer (4, 5).

Approximately 2,416 studies conducted in more than 200 countries/regions across the world have shown significant increase in the prevalence of overweight or obese people over the last four decades (from 25% in 1975 to 40% in 2014) (6). Over the last decade, the incidence of subclinical hypothyroidism increased from 3.2 to 16.7% (7). Although TSH levels are frequently above the upper limit of the normal range in overweight or obese individuals, it does not necessarily indicate thyroid dysfunction. Instead, it may be attributed to compensatory physiological mechanisms. Furthermore, pollutants, chemicals, and radiation in the environment may have an impact on thyroid function, leading to an increased incidence of subclinical hypothyroidism. A deficiency of trace elements such as selenium in the diet can also lead to thyroid dysfunction. With the changing dietary structure and increasing inadequacy of selenium supply in modern times, the incidence of subclinical hypothyroidism may also increase. The increased incidence of subclinical hypothyroidism is also related to increased chance of diagnosis. Hypothyroidism is the most common thyroid disorder in Chinese adults, particularly among women (7). Thyroid hormones play an essential role in lipid mobilization, lipid degradation, and fatty acid oxidation (8). The deficiency of thyroid hormones is associated with hyperlipidemia (9). In addition, patients with hypothyroidism develop insulin resistance and experience an average weight gain of 15%, both of which are significantly associated with differentiated thyroid cancer (10, 11). TSH levels are also closely related to the invasiveness and clinical stage of thyroid cancer (12, 13).

Regarding weight gain associated with subclinical hypothyroidism, there is controversy surrounding its role. Recent studies have shown that obesity is related to subclinical hypothyroidism, while others argue that the relationship is not significant (4, 5, 12–14). Whether the relationship between obesity and tumor invasiveness is mediated *via* subclinical hypothyroidism is not clear. In this study, our goal was to assess whether the relationship between subclinical hypothyroidism and PTC invasiveness depends on *body mass index.*

## Materials and methods

### Trial design, setting and participants

The retrospective cohort study included 13,717 patients with newly-diagnosed and operable PTC between 2008 and 2017. The study used a single-center consecutive sample at the Division of Thyroid Surgery of the China-Japan Union Hospital of Jilin University. This study was approved by the Health Care Ethics Committee of the China-Japan Union Hospital of Jilin University (No. 2019040806).

### Eligibility criteria

**Figure 1:**
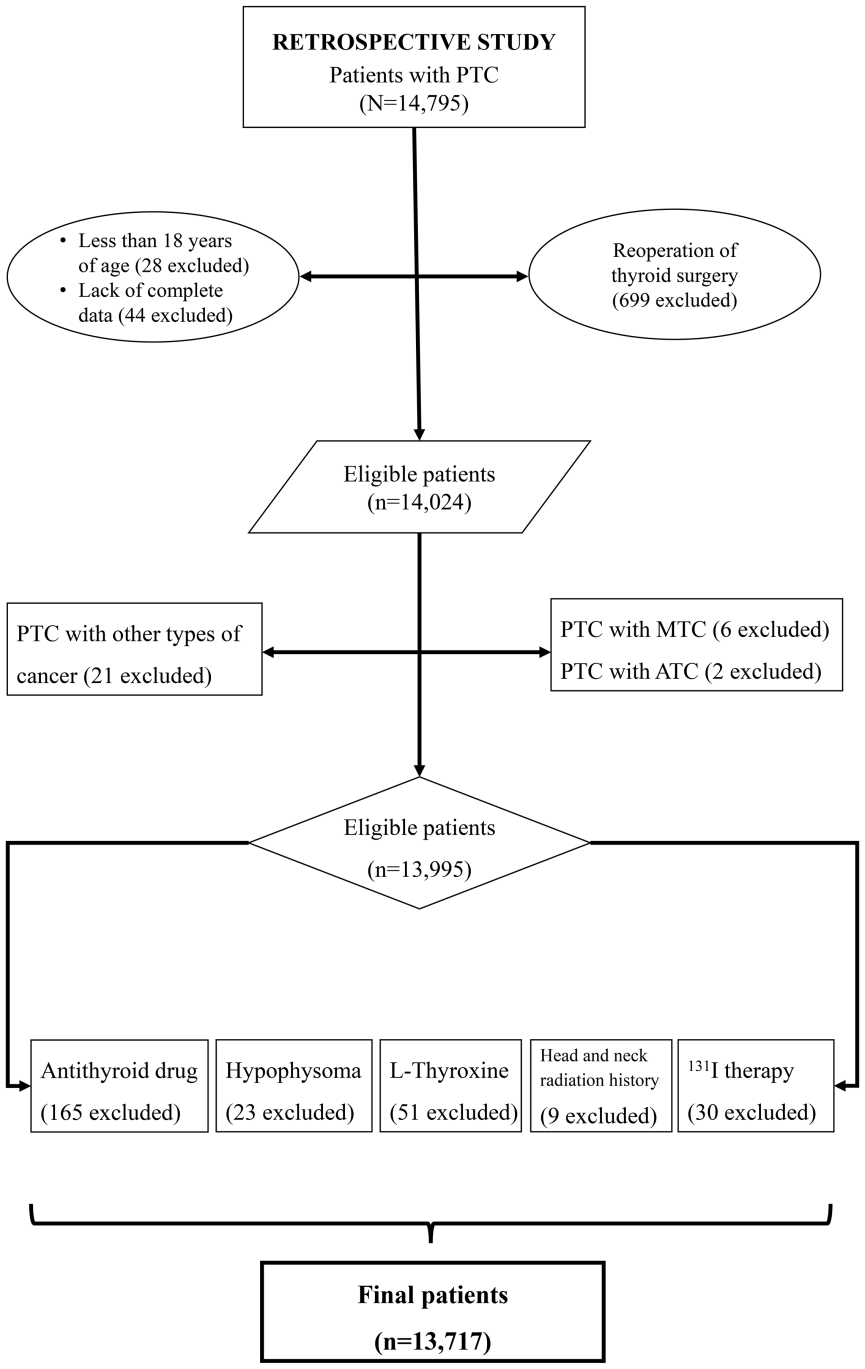
Flow chart of this study.

In retrospective study, the inclusion criteria: patients who underwent thyroid surgery for the first time; PTC confirmed through histopathological examination of surgical specimens; age >18 years; and absence of secondary cancers and/or other subtypes of thyroid cancer. As shown in **Figure 1**, 13,717 patients were included in the retrospective study.

### Data collection

After confirming the eligibility of patients, the data were collected using Microsoft Excel (Redmond, WA, USA). The data collection was standardized among physicians in this study. Standardized data collection includes the development and adherence to a protocol, the use of standardized tools, training and instruction, clear data recording procedures, and quality control measures to ensure consistency and reliability. The treating physicians were responsible for entering data about the patient, tumor, and treatment characteristics. Registry information was supplemented with data abstracted from medical records in the hospitals and physician offices. Basic data were recorded before surgery, including height, weight, and thyroid function tests. Patient age at the time of diagnosis was recorded. Thyroid function and thyroid antibodies tests, including levels of thyroid-stimulating hormone (TSH), free triiodothyronine (FT3), free thyroxine (FT4), thyroid peroxidase antibody (TPO-Ab), thyroglobulin (Tg), and thyroglobulin antibody (Tg-Ab), were also recorded. Pathological features, including the maximum primary tumor diameter, number of lesions, extrathyroidal extension (ETE), number of lymph node metastases (including central and lateral neck lymph node), presence of distant metastases, TNM stage, and clinical stage, were recorded. The pathologic stages of tumors were defined according to the American Joint Committee on Cancer (AJCC) staging criteria (15).

### Explanatory variables

#### Thyroid disease

As the time span of the included patients exceeded 10 years, multiple types of test kits were used during this period. Therefore, we defined thyroid dysfunction according to different kit reference standards at each stage. Subclinical hyperthyroidism was defined as TSH level below the reference range, with FT3 and FT4 levels within the normal reference range (16–18). Subclinical hypothyroidism was defined as TSH level above the reference range, with FT3 and FT4 in the normal range. Patients with TSH level below the reference range, FT3 and/or FT4 above the reference range were defined as having clinical hyperthyroidism. Patients with TSH level above the reference range, T3 and/or FT4 below the reference range were defined as having clinical hypothyroidisms. Cases of hypothyroidism were diagnosed when the patients received the thyroid cancer diagnosis. Thyroid ultrasound is a preoperative diagnostic tool that can aid in the detection of subclinical and clinical hypothyroidism. In the present research, this technique was also used as an auxiliary diagnosis to make a comprehensive judgment (**Supplementary Figure 1**).

#### Invasive tumor growth features

The invasive tumor growth features assessed included maximum tumor diameter > 1 cm, multifocality, ETE, presence of lymph node metastasis, a high number of lymph node metastases (> 5 by convention), high T stage (T3/T4), high clinical stage (III/IV), and distant metastasis (2).

#### Body mass index

The body mass index (BMI) was estimated using the body weight (kg) and height (m) measurements of patients obtained at the time of diagnosis; these data were retrieved from the physician records. BMI was defined in quartiles according to World Health Organization (WHO) standards, including underweight (< 18.5 kg/m^2^), normal-weight (18.5–24.9 kg/m^2^), overweight (25–29.9 kg/m^2^), and obese (≥ 30 kg/m^2^) (19).

### RNA extraction and quantitative real-time PCR

In this study, we utilized normal thyroid cells (Nthy-ori 3-1) and differentiated thyroid cancer cell lines (TPC-1, K-1, BCPAP, and KTC-1). Once the cells reached 80-90% confluence, we extracted RNA using TRIZOL reagent and further purified it using the GeneJET RNA purification kit. RNA concentrations were determined using a Thermo Scientific Multiskan Sky-high full-spectrum microplate reader. The reverse transcription reaction was conducted using the RevertAid first strand cDNA synthesis kit, with incubation at 42°C for 60 minutes, followed by heating to 70°C for 5 minutes. qRT-PCR was performed to detect the expression level of target genes. For this process, we used SYBR™Green Master Mix (5µl), forward and reverse primers (0.5µl each), cDNA (1µl), and H_2_O (3µl). By employing this experimental design and operation process, we aimed to minimize experimental errors and ensure the accuracy and reliability of the results.

### Outcomes and statistical analysis

The factors influencing subclinical hypothyroidism were further analyzed. The status of hypothyroidism at the time of thyroid cancer diagnosis was considered in the analysis. Continuous variables data are expressed as mean (standard deviation), while categorical variables are expressed as frequency (%). Continuous variables were analyzed using *t* test or analysis of variance, and categorical variables were analyzed using the Chi-squared test or Fischer’s exact test. Binary logistic regression was used to calculate the odds ratios (OR) and 95% confidence intervals (CI) of dependent variables. Thyroid function status and pathological characteristics were used as dependent variables. Statistical analysis was performed using SPSS 22.0 (IBM, Chicago, IL, USA).

### Bioinformatics analysis

We performed a comprehensive bioinformatics analysis using The Cancer Genome Atlas (TCGA), a publicly available database. Through Gene Ontology (GO), Gene Set Enrichment Analysis (GSEA), and Kyoto Encyclopedia of Genes and Genomes (KEGG) pathway analysis, we predicted the underlying mechanisms of thyroid cancer pathogenesis. Statistical analysis was conducted using R version 4.2.1 and the ggplot2 software package for data analysis and visualization. During the data preprocessing stage, we performed quality control on the raw data and identified genes with significant expression differences. Gene function annotation was carried out using GO, GSEA, and KEGG pathway analysis to enrich and annotate the identified genes. By comparing differentially expressed genes between normal and tumor tissue, we identified key genes and pathways associated with thyroid cancer development. To visually present our findings, we utilized the ggplot2 software package to create statistical graphs and visualize enrichment analysis results. In the differential expression analysis, a statistical significance threshold of p-value less than 0.05 was set, and genes with a log fold change (logFC) of ±1.5 were considered differentially expressed.

## Results

### Clinicopathological characteristics of eligible patients

As shown in **Table 1**, 13,717 PTC patients were included in the retrospective study. Approximately 78.5% (10,769/13,717) were women, with an average age (standard deviation) of 42.90 (9.43) years. The average BMI was 24.29 (3.48), with 58.1% being normal-weight (7,968/13,717) and 6.0% being obese (827/13,717). Tumor diameters > 1 cm were found in 22.7% of patients, multifocality in 40.4%, ETE in 26.7%, lymph node metastasis in 42.0%, high T stage in 2.7%, high clinical stage in 0.1%, and distant metastasis in 0.2%.

**Table 1:** Baseline characteristics of patients with papillary thyroid carcinoma (PTC).

Characteristic	Total (N=13,717)	Characteristic	Total (N=13,717)
	Mean (SD) or N (%)		Mean (SD) or N (%)
Sex		FT4 (pmol/L)	15.87 (5.85)
Female	10769 (78.5%)	T3 (nmol/L)	2.22 (2.04)
Male	2948 (21.5%)	T4 (nmol/L)	91.57 (15.89)
Mean age (years)	42.90 (9.43)	Tg (ng/mL)	19.14 (43.41)
Height (cm)	164.14 (6.92)	TPO-Ab (IU/mL)	47.75 (104.69)
Weight (kg)	65.73 (12.17)	TRAB (IU/L) ^b^	3.88 (1.46)
Mean BMI (kg/m^2^)	24.29 (3.48)	Tumor diameter >10 mm	3115 (22.7%)
BMI group ^a^		Multifocality	5535 (40.4%)
Underweight	432 (3.1%)	Extrathyroidal extension	3663 (26.7%)
Normal-weight	7968 (58.1%)	High T stage ^c^	371 (2.7%)
Overweight	4490 (32.7%)	Lymph node metastasis	5767 (42.0%)
Obese	827 (6%)	High number of lymph node metastases	1430 (10.4%)
Thyroid Function		High clinical stage ^c^	14 (0.1%)
TSH (mIU/L)	3.12 (2.98)	Distant metastasis	23 (0.2%)
FT3 (pmol/L)	4.54 (1.03)		

BMI, body mass index; TSH, thyroid stimulating hormone; FT3, free triiodothyronine; FT4, free thyroxine; T3, tri-iodothyronine; T4, thyroxine; TPO-Ab, thyroid peroxidase antibody; Tg , thyroglobulin; Tg-Ab, thyroglobulin antibody; TRAB, thyrotrophin receptor antibody.

a According to the World Health Organization (WHO‐BMI) definition.

b Results of 169 cases of hyperthyroidism.

c Determined by 8^th^ Edition of AJCC/UICC TNM Staging System.

### Thyroid dysfunction

As shown in **Table 2**, 11,759 patients (85.7%) had normal thyroid function, while 1,958 patients (12.3%) had abnormal thyroid function. Subclinical hyperthyroidism, subclinical hypothyroidism, hyperthyroidism, and hypothyroidism accounted for 1.2%, 11.1%, 0.4%, and 1.6% of patients, respectively (*P*<0.001).

**Table 2:** Thyroid dysfunction in men and women.

	Male n/N (%)	Female n/N (%)	Total n/N (%)	*P_male vs. female_*
Normal thyroid function	2724 (92.4%)	9035 (83.9%)	11759 (85.7%)	<0.001**
Subclinical hyperthyroidism	26 (0.9%)	143 (1.3%)	169 (1.2%)	0.017*
Subclinical hypothyroidism	171 (5.8%)	1347 (12.5%)	1518 (11.1%)	<0.001**
Hyperthyroidism	7 (0.2%)	44 (0.4%)	51 (0.4%)	0.111
Hypothyroidism	20 (0.7%)	200 (1.9%)	220 (1.6%)	<0.001**
*P value*	<0.001**	<0.001**	<0.001**	

* P<0.05, ** P<0.01.

### Thyroid dysfunction and invasive pathological features

The proportion of cases with ETE in the subclinical hypothyroidism group (29.3%) was higher than that in other groups [normal thyroid function group (26.7%), subclinical hyperthyroidism group (17.8%), hyperthyroidism group (19.6%), and hypothyroidism group (18.6%)] (**Table 3**). Binary logistic regression analysis showed that age, sex, weight, BMI, lgTPO-Ab, and lgTg-Ab were risk factors for ETE. The factors affecting ETE were used as confounding factors to adjust the OR value of the relationship between subclinical hypothyroidism and ETE (**Table 4**). As shown in **Table 5**, subclinical hypothyroidism was found to be an independent risk factor for ETE (OR adjusted, 1.168 [95% CI, 1.028–1.327]; *P*=0.017). The analysis of tumors with a diameter greater than 1 cm found that there is no association between the four different types of thyroid dysfunction and ETE (**Table 6**).

**Table 3:** Relationship between thyroid dysfunction and invasive pathological features.

	Normal thyroid function n/N (%)	Subclinical hyperthyroidism n/N (%)	Subclinical hypothyroidism n/N (%)	Hyperthyroidism n/N (%)	Hypothyroidism n/N (%)	*P value*
High tumor size >10mm	2640 (22.5%)	34 (20.1%)	369 (24.3%)	13 (25.5%)	59 (26.8%)	0.227
Multifocality	4752 (40.4%)	77 (45.6%)	604 (39.8%)	18 (35.3%)	84 (38.2%)	0.542
Extrathyroidal extension	3137 (26.7%)	30 (17.8%)	445 (29.3%)	10 (19.6%)	41 (18.6%)	<0.001^**^
High T stage	309 (2.6%)	5 (3.0%)	45 (3.0%)	3 (5.9%)	9 (4.1%)	0.372
Lymph node metastasis	4985 (42.4%)	58 (34.3%)	605 (39.9%)	20 (39.2%)	99 (45%)	0.070
High number of lymph node metastases	1233 (10.5%)	15 (8.9%)	149 (9.8%)	5 (9.8%)	28 (12.7%)	0.671
High clinical stage	10 (0.1%)	1 (0.6%)	3 (0.2%)	0 (0%)	0 (0%)	0.203
Distant metastasis	20 (0.2%)	1 (0.6%)	2 (0.1%)	0 (0%)	0 (0%)	0.664

** P<0.01.

**Table 4:** Results of binary logistic regression analysis showing factors influencing extrathyroidal extension.

Characteristic	Extra thyroidal extension	
	Crude OR (95% CI)	*P* value
Age	1.011 (1.007-1.016)	<0.001**
Sex	1.163 (1.063-1.273)	0.001**
Weight	1.009 (1.006-1.012)	<0.001**
Height	1.003 (0.998-1.009)	0.221
BMI	1.036 (1.025-1.047)	<0.001**
lgTSH	1.310 (1.163-1.477)	<0.001**
lgFT3	2.934 (1.797-4.79)	<0.001**
lgFT4	1.611 (0.93-2.79)	0.089
lgTPO-Ab	0.874 (0.809-0.944)	0.001**
lgTg-Ab	1.268 (1.195-1.345)	<0.001**
Hyperthyroidism	0.670 (0.335-1.34)	0.258
Subclinical hyperthyroidism	0.593 (0.399-0.882)	0.010*
Hypothyroidism	0.630 (0.447-0.886)	0.008**
Subclinical hypothyroidism	1.140 (1.013-1.282)	0.029*

BMI, body mass index; TSH, thyroid stimulating hormone; FT3, free triiodothyronine; FT4, free thyroxine; TPO-Ab, thyroid peroxidase antibody; Tg-Ab, thyroglobulin antibody; OR, odds ratio.

**P<0.01.

**Table 5:** Binary logistic regression analysis showing relationship between thyroid dysfunction and extrathyroidal extension.

	Crude OR ^a^ (95% CI)	*P value*	Adjusted OR ^b^ (95% CI)	*P value*
Subclinical hyperthyroidism	0.593 (0.399-0.882)	0.010^*^	0.606 (0.391-0.938)	0.025^*^
Subclinical hypothyroidism	1.140 (1.013-1.282)	0.029^*^	1.168 (1.028-1.327)	0.017^*^
Hyperthyroidism	0.670 (0.335-1.34)	0.258	0.861 (0.422-1.754)	0.679
Hypothyroidism	0.630 (0.447-0.886)	0.008^**^	0.668 (0.459-0.973)	0.035^*^

OR, odds ratio.

a Logistic regression crude OR included thyroid dysfunction as covariates.

b Logistic regression adjusted OR included thyroid dysfunction, age, sex, weight, BMI, lgTPO-Ab, and lgTg-Ab as covariates.

^*^ P<0.05, ^**^ P<0.01.

**Table 6:** Binary logistic regression analysis showing relationship between thyroid dysfunction and extrathyroidal extension in cases greater than 1.0 cm.

	Crude OR ^a^ (95% CI)	*P value*	Adjusted OR ^b^ (95% CI)	*P value*
Subclinical hyperthyroidism	0.562 (0.273-1.157)	0.118	0.568 (0.252-1.282)	0.173
Subclinical hypothyroidism	1.181 (0.950-1.469)	0.135	1.215 (0.958-1.540)	0.107
Hyperthyroidism	0.522 (0.160-1.699)	0.280	0.651 (0.185-2.288)	0.504
Hypothyroidism	0.649 (0.379-1.112)	0.116	0.708 (0.389-1.291)	0.261

OR, odds ratio.

a Logistic regression crude OR included thyroid dysfunction as covariates.

b Logistic regression adjusted OR included thyroid dysfunction, age, sex, weight, BMI, lgTPO-Ab, and lgTg-Ab as covariates.

### Different BMI subgroups, subclinical hypothyroidism, and ETE

As shown in **Table 7**, subclinical hypothyroidism in the normal-weight group was an independent risk factor for ETE (OR adjusted, 1.287 [95% CI, 1.089–1.520]; *P*=0.003). Subclinical hypothyroidism was not associated with ETE in under-weight, overweight, and obese groups (*P1 =* 0.389; *P2 =* 0.954; *P3 =* 0.241, respectively).

**Table 7:** Relationship between subclinical hypothyroidism and extrathyroidal extension in different BMI subgroups.

WHO-BMI	Crude OR ^a^ (95% CI)	*P value*	Adjusted OR ^b^ (95% CI)	*P value*
Underweight (<18.5)	0.934 (0.431-2.026)	0.864	0.673 (0.274-1.655)	0.389
Normal-weight (18.5‐24.9)	1.217 (1.042-1.42)	0.013^*^	1.287 (1.089-1.520)	0.003^**^
Overweight (25‐29.9)	0.998 (0.81-1.229)	0.982	0.993 (0.791-1.247)	0.954
Obese (≥30)	1.301 (0.84-2.013)	0.238	1.327 (0.827-2.128)	0.241

OR, odds ratio.

a Logistic regression crude OR included thyroid dysfunction as covariates.

b Logistic regression adjusted OR included thyroid dysfunction, age, sex, weight, lgTPO-Ab, and lgTg-Ab as covariates.

*P<0.05, **P<0.01.

### Different gender subgroups, subclinical hypothyroidism, and ETE

Binary logistic regression was used to compare the difference between subclinical hypothyroidism and ETE in man and woman subgroups in different BMI groups. The results showed that in normal-weight PTC, subclinical hypothyroidism was a risk factor for ETE irrespective of the sex (OR1 adjusted, 2.363 [95% CI, 1.273–4.386], *P*=0.006; OR2 adjusted, 1.228 [95% CI, 1.032–1.462], *P*=0.021). Compared to females, subclinical hypothyroidism was a higher risk factor for ETE in male patients with normal body weight (OR male adjusted, 2.363 [95% CI, 1.273–4.386], *P*=0.006; OR female adjusted, 1.228 [95% CI, 1.032–1.462], *P*=0.021). Similar trends are observed in underweight, overweight, and obesity patients (**Table 8**).

**Table 8:** Relationship between subclinical hypothyroidism and extrathyroidal extension in gender subgroups.

WHO-BMI	Male		Female	
	Adjusted OR ^a^ (95% CI)	*P value*	Adjusted OR ^a^ (95% CI)	*P value*
Underweight (<18.5)	3.390 (0.364-31.56)	0.284	0.533 (0.203-1.403)	0.203
Normal-weight (18.5‐24.9)	2.363 (1.273-4.386)	0.006^**^	1.228 (1.032-1.462)	0.021^*^
Overweight (25‐29.9)	1.448 (0.901-2.328)	0.126	0.895 (0.69-1.161)	0.403
Obese (≥30)	1.692 (0.726-3.942)	0.223	1.188 (0.67-2.109)	0.555

OR, odds ratio.

a Logistic regression crude OR included thyroid dysfunction as covariates.

b Logistic regression adjusted OR included thyroid dysfunction, age, sex, weight, BMI, lgTPO-Ab, and lgTg-Ab as covariates.

^*^P<0.05, ^**^P<0.01.

### Different age subgroups, subclinical hypothyroidism, and ETE

Binary logistic regression was used to compare the relationship between subclinical hypothyroidism and ETE in different age groups. The age cut-point value was set from 35 to 55 years old, as shown in **Table 9**. The cut point for subclinical hypothyroidism and ETE was 38 years old. The results showed that in the less than 38 years old subgroup, subclinical hypothyroidism was a risk factor for ETE (OR1 adjusted, 1.382 [95% CI, 1.032–1.852], *P*=0.030). However, in the more than 38 years old subgroup, subclinical hypothyroidism was not associated with ETE (*P*=0.054) (**Table 9**).

**Table 9:** Relationship between subclinical hypothyroidism and extrathyroidal extension in age subgroups.

Age cut-point	Adjusted OR ^a^ (95%CI)	*P value*	Adjusted OR ^a^ (95%CI)	*P value*
**35 years old**	*≤35*		*>35*	
	1.412 (0.987-2.022)	0.059	1.238 (1.024-1.496)	0.027^*^
**36 years old**	*≤36*		*>36*	
	1.357 (0.974-1.89)	0.071	1.244 (1.024-1.511)	0.028^*^
**37 years old**	*≤37*		*>37*	
	1.342 (0.98-1.838)	0.067	1.241 (1.018-1.513)	0.032^*^
**38 years old**	*≤38*		*>38*	
	1.382 (1.032-1.852)	0.030^*^	1.222 (0.996-1.5)	0.054
**39 years old**	*≤39*		*>39*	
	1.458 (1.108-1.92)	0.007^**^	1.173 (0.95-1.449)	0.138
**40 years old**	*≤40*		*>40*	
	1.415 (1.088-1.839)	0.010^*^	1.174 (0.944-1.46)	0.148
**41 years old**	*≤41*		*>41*	
	1.325 (1.028-1.708)	0.030^*^	1.229 (0.983-1.536)	0.070
**42 years old**	*≤42*		*>42*	
	1.351 (1.059-1.723)	0.015^*^	1.202 (0.954-1.514)	0.119
**43 years old**	*≤43*		*>43*	
	1.33 (1.052-1.681)	0.017^*^	1.216 (0.958-1.544)	0.109
**44 years old**	*≤44*		*>44*	
	1.341 (1.072-1.679)	0.010^*^	1.196 (0.93-1.537)	0.162
**45 years old**	*≤45*		*>45*	
	1.336 (1.076-1.659)	0.009^**^	1.198 (0.92-1.559)	0.181
**50 years old**	*≤50*		*>50*	
	1.239 (1.024-1.501)	0.028^*^	1.416 (1-2.004)	0.050
**55 years old**	*≤55*		*>55*	
	1.275 (1.069-1.521)	0.007^**^	1.328 (0.769-2.294)	0.308

OR, odds ratio.

a Logistic regression adjusted OR included thyroid dysfunction, age, sex, weight, BMI, lgTPO-Ab, and lgTg-Ab as covariates.

* P<0.05, ** P<0.01.

### Different BMI subgroups, TPO-Ab, Tg-Ab and ETE

We analyzed the relationship between TPO-Ab, Tg-Ab, and ETE in different BMI groups. As shown in **Table 10**, in the normal weight and overweight subgroups, TPO-Ab was a protective factor for ETE (OR1 adjusted, 0.734 [95% CI, 0.653-0.824], *P*<0.001); OR2 adjusted, 0.814 [95% CI, 0.692-0.957], *P*=0.013). TPO-Ab was not associated with ETE in the underweight, overweight, and obese groups (*P*=0.883 for underweight, *P*=0.405 for overweight). In all BMI subgroups, Tg-Ab was a risk factor for ETE (OR1 adjusted, 1.585 [95% CI, 1.120-2.243], *P*=0.009; OR2 adjusted, 1.424 [95% CI, 1.306-1.551], *P*<0.001; OR3 adjusted, 1.378 [95% CI, 1.226-1.549], *P*<0.001; OR4 adjusted, 1.465 [95% CI, 1.124-1.911], *P=*0.005).

**Table 10:** Relationship between TPO-Ab、Tg-Ab and extrathyroidal extension in different BMI subgroups.

	WHO-BMI	Crude OR ^a^ (95% CI)	*P value*	Adjusted OR ^b^ (95% CI)	*P value*
TPO-Ab	Underweight	1.224 (0.819-1.83)	0.324	0.964 (0.587-1.582)	0.883
	Normal-weight	0.841 (0.760-0.930)	<0.001**	0.734 (0.653-0.824)	<0.001**
	Overweight	0.926 (0.807-1.064)	0.278	0.814 (0.692-0.957)	0.013*
	Obese	0.879 (0.635-1.216)	0.436	0.942 (0.818-1.085)	0.405
Tg-Ab	Underweight	1.623 (1.19-2.215)	0.002**	1.585 (1.12-2.243)	0.009**
	Normal-weight	1.272 (1.177-1.375)	<0.001**	1.424 (1.306-1.551)	<0.001**
	Overweight	1.256 (1.130-1.397)	<0.001**	1.378 (1.226-1.549)	<0.001**
	Obese	1.312 (1.044-1.65)	0.02*	1.465 (1.124-1.911)	0.005**

OR, odds ratio.

a Logistic regression crude OR included thyroid dysfunction as covariates.

b Logistic regression adjusted OR included thyroid dysfunction, age, sex, weight, and lgTPO-Ab/lgTg-Ab as covariates.

*P<0.05, **P<0.01.

### Subclinical hypothyroidism and ultrasonic characteristics

In further analysis, we compared the differences in ultrasound features between the normal thyroid function and subclinical hypothyroidism groups in terms of components, echoes, shape, margins, and strong echoic focus. The results revealed that the proportion of longitudinal-to-transverse ratio > 1 was lower in the subclinical hypothyroidism group compared to the normal thyroid function group (13.2% vs. 15.5%, *P*=0.023). However, there were no statistically significant differences observed in other ultrasound features, including Components, Echoes, Margins, and Strong echoic focus (**Supplementary Table 1**).

### Thyrotropin receptor and clinical features

The thyrotropin receptor (TSHR) is a membrane-bound receptor found on the surface of thyroid cells. It plays a crucial role in regulating the function and growth of the thyroid gland. TSHR binds to TSH, which is released by the pituitary gland, and initiates a signaling cascade that leads to the production and release of thyroid hormones. Understanding the mechanisms and functions of the TSHR is vital for diagnosing and treating thyroid-related conditions.

We analyzed the expression of TSHR in thyroid cancer tissues using the TCGA database. The results revealed that the expression level of TSHR gene was lower in thyroid cancer tissues compared to normal tissues. There was no significant difference in TSHR expression between thyroid cancer tissues and lymphocyte infiltrated tissues (**Supplementary Figure 2**).

We examined the relationship between TSHR expression and T stage, N stage, ETE, pathologic stage, and prognosis. The results showed a negative correlation between TSHR expression and invasive features, including advanced T stage, lymph node metastasis, ETE, and higher pathologic stage. Moreover, lower TSHR expression was associated with a poorer platinum free interval (PFI) (**Supplementary Figure 3**).

### Functional enrichment and pathway analysis of TSHR-associated genes

The TCGA database were analyzed for differential gene expression between cases with ETE and those without ETE. The results showed significant variations in gene expression between the two groups, with logFC values ranging from ±1.5 and *P*-values below 0.05 (**Supplementary Figure 4A**). Among the differentially expressed genes, 529 genes overlapped with those associated with thyroid-stimulating hormone receptor (TSHR) and epithelial-mesenchymal transition (EMT) (**Supplementary Figure 4B**). KEGG enrichment analysis revealed that the autophagy, MAPK, and PI3K-Akt pathways were potentially involved in TSHR/EMT/ETE (**Supplementary Figures 4C, D**). Further analysis combining KEGG pathway with logFC ± 1.5 indicated that the autophagy pathway was most likely involved (**Supplementary Figure 4E**). In-depth functional exploration of these genes was conducted through enrichment analysis using the GSEA database, it revealed a negative correlation between the thyroid autoimmune pathway and the autophagy pathway (**Supplementary Figure 4F**).

### Verification of gene expression levels in the deregulated pathway of thyroid carcinomas

As shown in **Supplementary Figure 5A**, there was a significant difference in the expression level of the TSHR gene between normal thyroid cells and differentiated thyroid cancer cells. The K-1 cell line displayed significantly lower TSHR expression levels compared to other cell lines. Therefore, we chose the K-1 cell line as the target for subsequent studies. To investigate the effect of TSH on autophagy in the K-1 cell line, we treated the cells with different concentrations of human recombinant TSH, including 5, 25, 50, 75, and 100 mU/ml. Subsequently, we examined changes in the expression of autophagy-related genes, including MAP1LC3B, p62, ULK-1, Beclin-1, ATG3, ATG5, and ATG7. The experimental results showed that the expressions of MAP1LC3B, Ulk1, ATG5 and ATG7 were significantly up-regulated, while the expression of p62 was significantly down-regulated. The expression of these genes showed a trend of concentration-dependent change, indicating that TSH had a concentration-dependent effect on the expression of autophagy-related genes (**Supplementary Figures 5B–H**).

## Discussion

**Figure 2:**
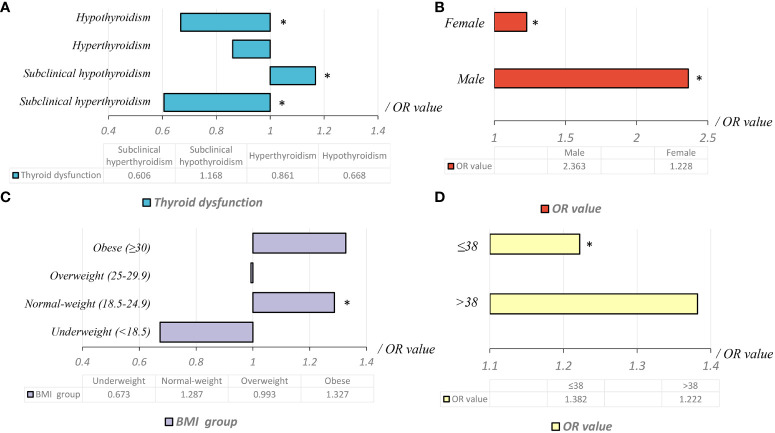
The relationship between subclinical hypothyroidism and invasive papillary thyroid cancer.

In the retrospective study that included 13,717 patients with PTC, subclinical hypothyroidism was an independent risk factor for ETE. In normal-weight patients, subclinical hypothyroidism was an independent risk factor for ETE. However, this risk was not observed in under-weight, overweight, and obese patients. The relationship between subclinical hypothyroidism and ETE was dependent on normal BMI and age, but not limited to gender (**Figure 2**).

Studies conducted in Spain and Australia have reported incidence of subclinical hypothyroidism of 4.6% and 5.0%, respectively (20, 21). The incidence of subclinical hypothyroidism in Japan was 8.9% (22). In India, a recent study found the incidence of subclinical hypothyroidism to be up to 10% (23). A large-scale study in China showed that the incidence of subclinical hypothyroidism was as high as 16.7% (7). In the current study, the incidence of subclinical hypothyroidism in PTC patients was 11.1%, which is similar to those mentioned above. One possible explanation for the higher incidence in China could be differences in population demographics and genetic factors. China has a significantly larger population compared to most Western countries, and factors such as iodine deficiency in certain regions of China, can contribute to an increased risk of thyroid disorders. Moreover, variations in healthcare practices and diagnostic criteria may also play a role. The diagnostic guidelines and thresholds for defining subclinical hypothyroidism might differ between China and Western countries, leading to variations in reported incidence rates. Cultural and environmental factors, such as dietary habits or exposure to certain pollutants, could also contribute to these differences.

The factors influencing subclinical hypothyroidism have not been fully elucidated. Studies have found that obesity is a risk factor for subclinical hypothyroidism. In a cross-sectional study of 2,808 Chinese patients, Wang et al. reported that obese women were at a significantly higher risk of developing subclinical hypothyroidism compared to non-obese women. The study found that the prevalence of subclinical hypothyroidism was 22.1% in obese women, while it was 13.4% in non-obese women (OR=1.83, 95%CI 1.20–2.80, *P* = 0.005). This finding suggests that obesity may be a contributing factor to the development of subclinical hypothyroidism in women (4). In the current study, obesity (BMI>30) was an independent risk factor for subclinical hypothyroidism (OR adjusted, 1.436; *P*=0.002). However, there is no clear consensus on the relationship between obesity and subclinical hypothyroidism. In a study by Ittermann et al., obesity was not found to increase the risk of hypothyroidism or subclinical hypothyroidism (24, 25).

In addition, excess iodine may be another risk factor associated with subclinical hypothyroidism. Yao et al. observed significant differences in the detection rates of clinical hypothyroidism across different iodine nutritional states. Particularly, the positive rate of hypothyroidism was higher in the iodine deficiency group compared to other iodine nutrition groups (26). The meta-analysis presented robust epidemiological evidence supporting the association between hypothyroidism and non-alcoholic fatty liver disease (NAFLD). Specifically, subclinical hypothyroidism was found to be significantly associated with NAFLD, (OR=1.40, 95% CI 1.10-1.77, *P* = 0.006) (27). Teng et al. (year) explored the association between thyroid function and metabolic syndrome and observed that the subclinical hypothyroidism group had higher levels of BMI, waist circumference, SBP, and TG compared to the normal thyroid function group (28).

Patients with subclinical hypothyroidism only showed abnormally elevated serum TSH levels, while thyroid hormone levels were normal. A recent meta-analysis of 56 studies confirmed that higher serum TSH levels were closely related to the size of thyroid tumors and lymph node metastasis (12). The systematic review and meta-analysis identified that Hashimoto’s thyroiditis was not found to be associated with lymph node metastasis in PTC (29). In this study, we found a significant association between subclinical hypothyroidism and ETE (OR=1.070, *P*=0.025). We also found that the correlation between subclinical hypothyroidism and ETE was dependent on body weight. Only in patients with normal weight, subclinical hypothyroidism (high levels of TSH) was associated with ETE (OR adjusted=1.113, *P*=0.007). In overweight/obese patients, subclinical hypothyroidism (high levels of TSH) was not associated with ETE. We have reasons to believe that this may be related to the physiological adaptation of the body. This suggests that elevated TSH in obese patients may be an adaptive response to weight gain mediated through the hypothalamic-pituitary-thyroid axis. In addition, elderly people may develop elevated levels of serum TSH due to physiological adaptation. Elevated serum TSH is a primary feature of thyroid failure.

In 2019, Cooper et al. put forward a view that patients with elevated serum TSH levels and no thyroid-related diseases or extreme obesity can only be referred to as having elevated TSH levels, rather than subclinical hypothyroidism in the true sense (30). In this study, we found that elevated serum TSH was not related to ETE in overweight/obese patients with PTC. It is possible that elevated serum TSH in obese patients was due to the adaptive physiological response of the body, a reversible change in thyroid function. For normal-weight individuals, elevated serum TSH may be caused by pathological changes in thyroid function. The pathological changes may be caused by certain genetic factors, environmental factors, pharmaceutical agents, or other clinical conditions. Therefore, thyroid cancer cells are more active and aggressive in this pathological state.

The MAPK signaling pathway is one of the common signaling pathways involved in the progression of PTC. Studies have shown that certain exogenous substances may exert anticancer effects through the MAPK signaling pathway (31). In this study, we found that autophagy may be another important mechanism in the progression of PTC. The results of GSEA and KEGG enrichment analysis demonstrate the involvement of the autophagy signaling pathway in regulating TSHR/ETE/EMT in thyroid cancer. Autophagy plays a crucial role in maintaining cellular homeostasis by eliminating damaged proteins and organelles, thereby ensuring cell survival and preventing the development of various diseases, including cancer. The identification of the autophagy pathway in our study adds to the growing body of evidence supporting its significance in thyroid cancer progression.

Interestingly, our findings also reveal a concentration-dependent relationship between TSH intervention level and the expression of key genes involved in the autophagy pathway. This suggests that TSH may exert its effects on thyroid cancer cells by modulating autophagy-mediated processes. Previous studies have implicated TSH in promoting tumor growth and metastasis in thyroid cancer, but the underlying mechanisms remain poorly understood (32–34). Our research provides valuable insights into the potential role of autophagy as a mediator of TSH signaling in thyroid cancer cells.

Furthermore, the autophagy signaling pathway has been extensively studied for its involvement in various cellular processes, including apoptosis, angiogenesis, and immune responses (35). Dysregulation of autophagy has been linked to the development and progression of several types of cancer, highlighting its potential as a therapeutic target (36). Therefore, our findings not only contribute to the understanding of TSHR/ETE/EMT regulation in thyroid cancer but also provide a rationale for exploring the targeting of autophagy as a novel therapeutic strategy for this disease.

In conclusion, our study demonstrates the involvement of the autophagy signaling pathway in regulating TSHR/ETE/EMT in thyroid cancer cells. The concentration-dependent relationship between TSH intervention levels and the expression of key genes in the autophagy pathway suggests a potential mechanistic link between TSH signaling and autophagy-mediated processes. Given the critical roles of autophagy in cancer development and the emerging evidence of TSH’s effects on tumor progression, future research should aim to unravel the precise molecular mechanisms underlying the interplay between TSH signaling, autophagy, and thyroid cancer. Such insights may pave the way for the development of innovative therapeutic approaches targeting autophagy for improved treatment outcomes in thyroid cancer patients.

## Limitations

This study was a relatively large single-center retrospective of the relationship between BMI subclinical hypothyroidism, and tumor invasiveness. However, certain limitations of the study should be acknowledged. Due to multiple factors such as patient requirements and medical policies, it was not possible to determine whether obese patients with PTC who have subclinical hypothyroidism experienced different tumor characteristics (e.g., invasiveness) after achieving weight loss through diet/exercise. In the case of altered TSH, the test was not repeated to confirm thyroid dysfunction. Given that the time span of the included patients exceeded 10 years, multiple types of test kits were used during this period. Consequently, we defined thyroid dysfunction based on different kit reference standards at each stage, instead of relying solely on a cut-off value for TSH determination.

In summary, our findings may help frontline clinicians to develop more personalized diagnoses and treatment plans for PTC patients with subclinical hypothyroidism. For normal-weight patients with PTC and subclinical hypothyroidism, a more active treatment plan should be adopted, with more cautious preoperative evaluation, more effective intraoperative strategy, and more frequent postoperative follow-up.

## Data availability statement

The original contributions presented in the study are included in the article/**Supplementary Material**, further inquiries can be directed to the corresponding author.

## Ethics statement

The studies involving humans were approved by the Health Care Ethics Committee of the China-Japan Union Hospital of Jilin University. The studies were conducted in accordance with the local legislation and institutional requirements. Written informed consent for participation was not required from the participants or the participants’ legal guardians/next of kin in accordance with the national legislation and institutional requirements.

## Author contributions

CL: Writing – original draft. JZ: Writing – review & editing. GD: Writing – review & editing. HS: Supervision, Writing – review & editing.
