# Supplementary material for: The relationship between subclinical hypothyroidism and invasive papillary thyroid cancer
Source: Front Endocrinol (Lausanne). 2023 Dec 20;14:1294441. doi: 10.3389/fendo.2023.1294441 (PMC10761496; doi:10.3389/fendo.2023.1294441)

**Supplement Figure 1.** *Ultrasound characteristics of papillary thyroid carcinoma complicated with subclinical hypothyroidism*


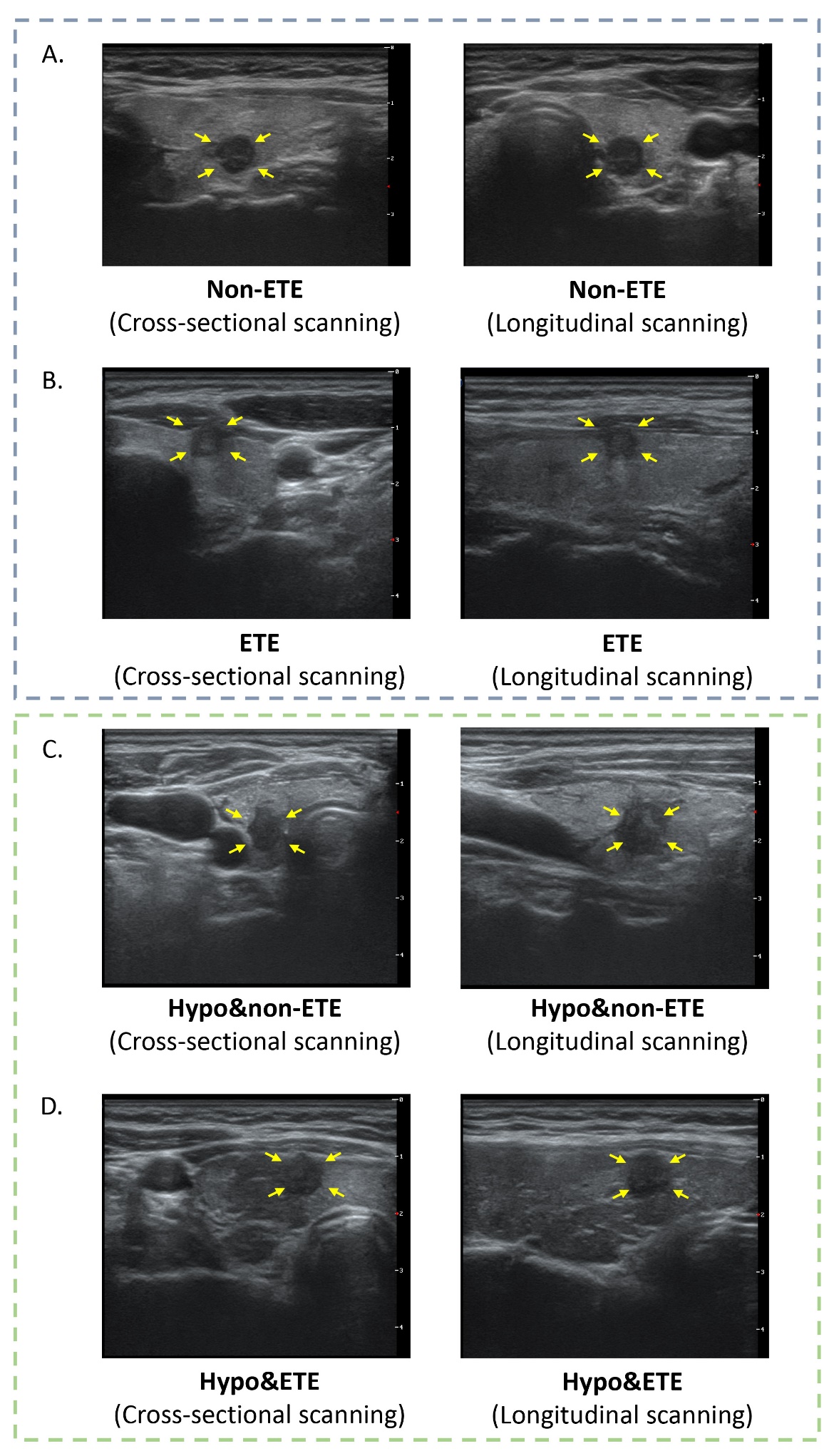


**Supplement Figure 2.** *Thyrotropin receptor (TSHR) expression level and immune infiltration in TCGA database*


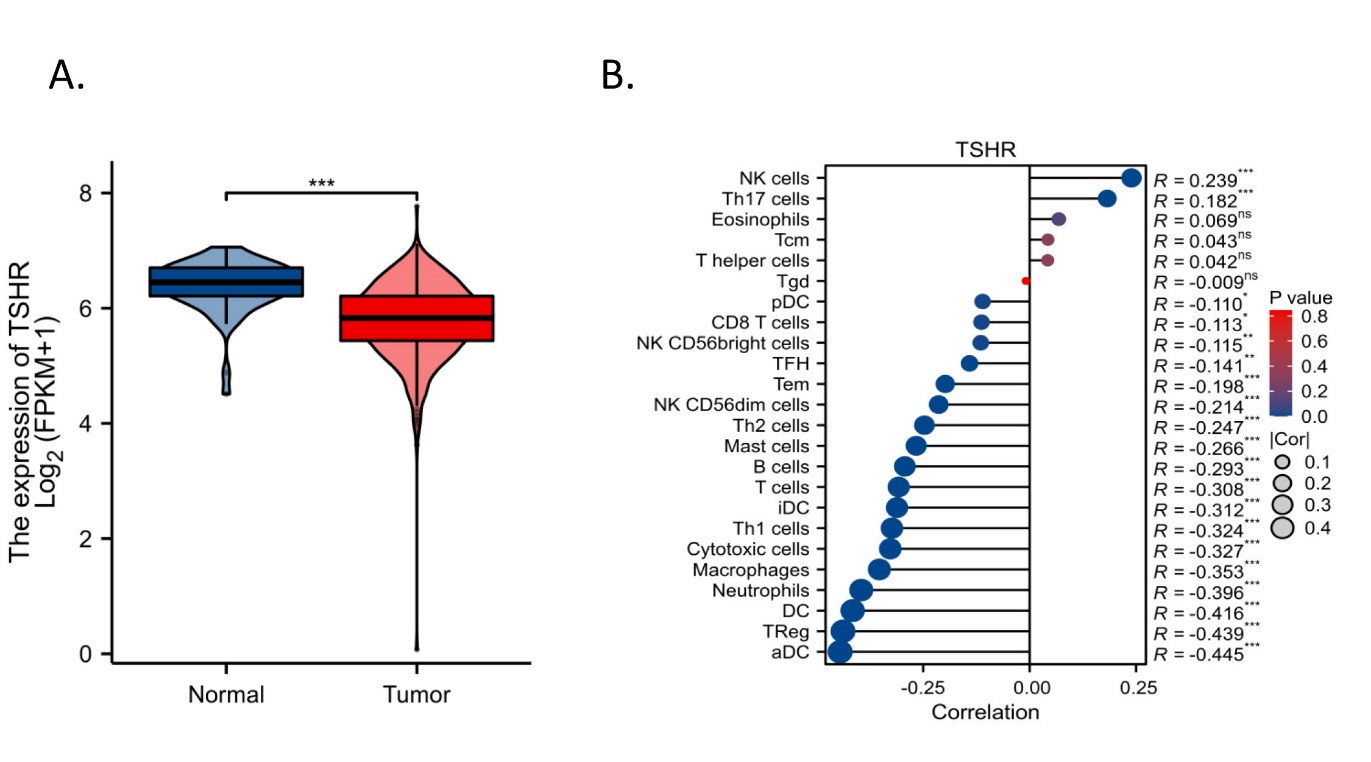


**Supplement Figure 3.** *The relationship between thyrotropin receptor (TSHR) and clinical features in TCGA database.*


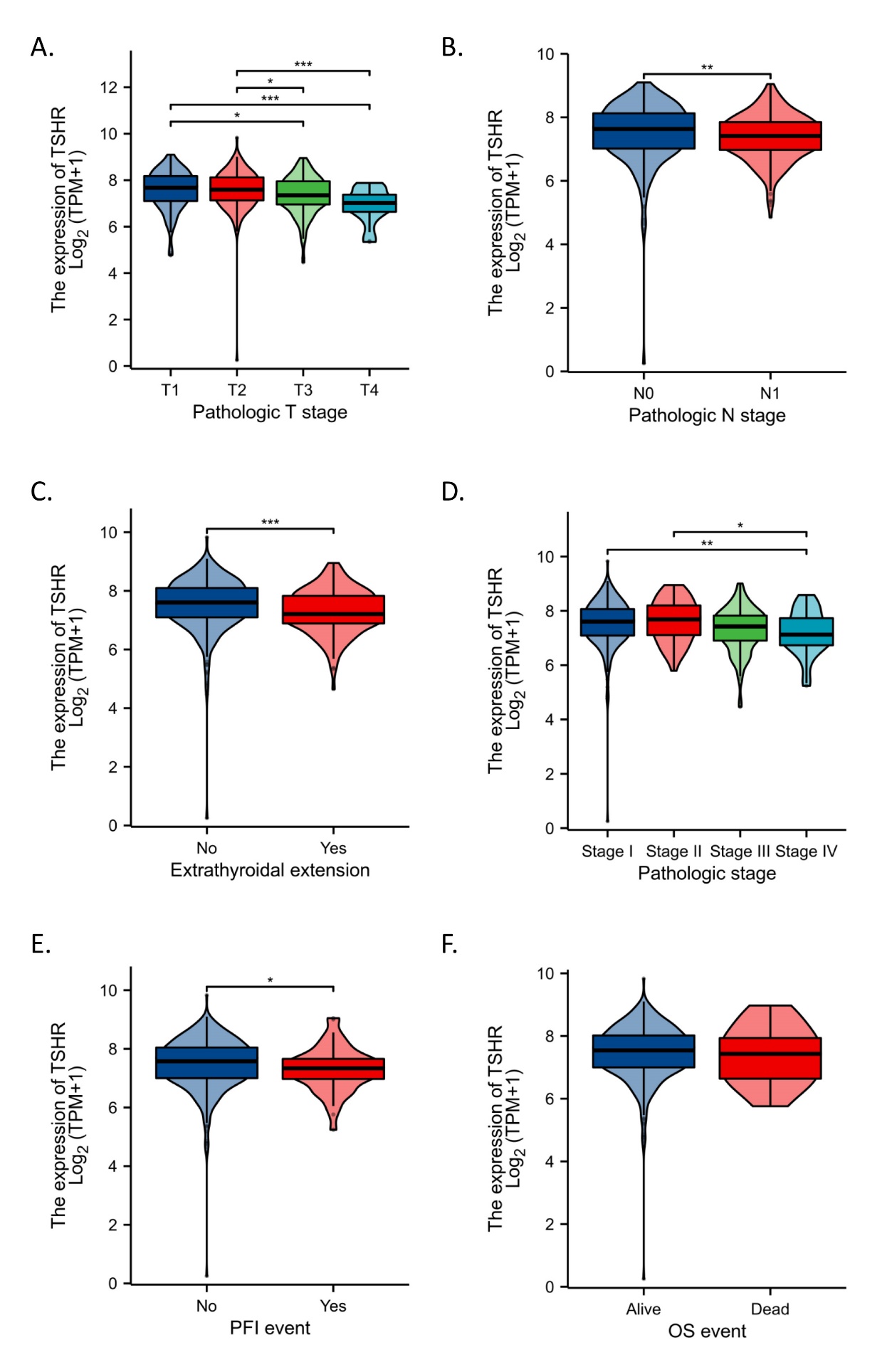


**Supplement Figure 4.** Functional enrichment analysis and KEGG pathway analysis of TSHR-related genes.


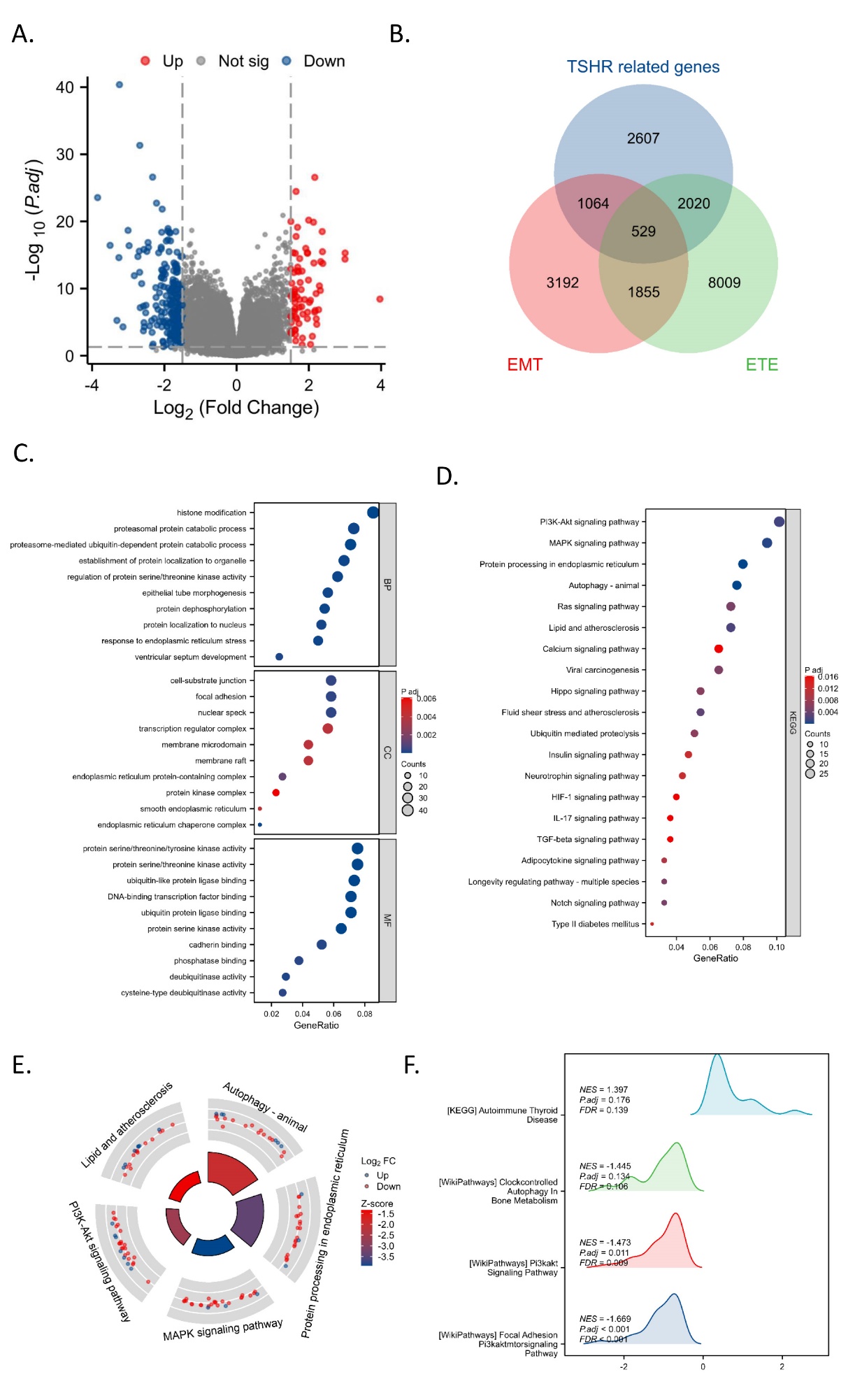
**Supplement Figure 5.** Verification of gene expression levels of pathway in thyroid cancer.


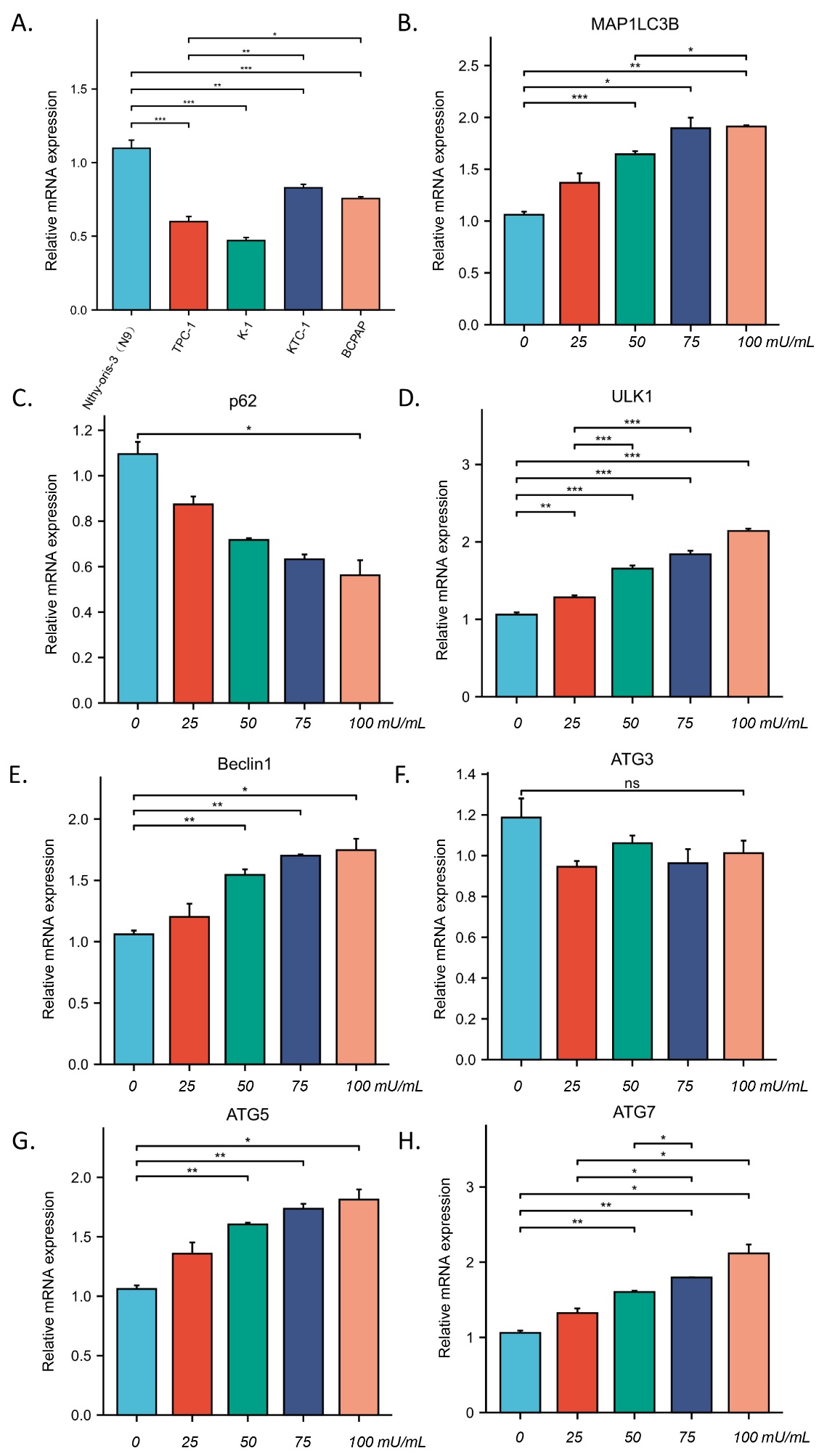

Supplement: Supplementary file 1 [file DataSheet_1.docx]
