# Supplementary material for: The relationship between subclinical hypothyroidism and invasive papillary thyroid cancer
Source: Front Endocrinol (Lausanne). 2023 Dec 20;14:1294441. doi: 10.3389/fendo.2023.1294441 (PMC10761496; doi:10.3389/fendo.2023.1294441)
Supplement: Supplementary file 2 [file Table_1.docx]

**Supplement Table 1** Relationship between ultrasonic characteristics and Subclinical hypothyroidism.

|  | Normal thyroid function  n/N (%) | *Subclinical hypothyroidism*  n/N (%) | *P* value |
| --- | --- | --- | --- |
| **Components** |  |  |  |
| Cystic or almost completely cystic | 1 (0%) | 0 (0%) | 0.233 |
| Spongy (occupying more than 50%) | 1071 (9.1%) | 115 (7.6%) |  |
| Mixed cystic and solid (dominated by solid and cystic) | 10685 (90.9%) | 1403 (92.4%) |  |
| Solid or almost completely solid | 2 (0%) | 0 (0%) |  |
| **Echoes** |  |  |  |
| No echo | 89 (0.8%) | 8 (0.5%) | 0.794 |
| Iso-/hyperechoic | 59 (0.5%) | 7 (0.5%) |  |
| Hypoechoic | 11043 (93.9%) | 1430 (94.2%) |  |
| **Shape** | 568 (4.8%) | 73 (4.8%) |  |
| Longitudinal-to-transverse ratio > 1 | 1817 (15.5%) | 201 (13.2%) | 0.023^*^ |
| Longitudinal-to-transverse ratio < 1 | 9942 (84.5%) | 1317 (86.8%) |  |
| **Margins** |  |  |  |
| Smooth | 8016 (68.2%) | 1004 (66.1%) | 0.260 |
| Unclear borders | 3384 (28.8%) | 462 (30.4%) |  |
| Lobulated or irregular | 359 (3.1%) | 52 (3.4%) |  |
| Extrathyroidal invasion | 568 (4.8%) | 73 (4.8%) |  |
| **Strong echoic focus** |  |  |  |
| No calcification or with comet tail artifact | 5331 (45.3%) | 712 (46.9%) | 0.707 |
| Coarse calcification | 2343 (19.9%) | 297 (19.6%) |  |
| Peripheral calcification | 117 (1%) | 15 (1%) |  |
| Dot-like strong echoes (including small comet tail artifacts) | 3968 (33.7%) | 494 (32.5%) |  |
